# Sense Transgene-Induced Post-Transcriptional Gene Silencing in Tobacco Compromises the Splicing of Endogenous Counterpart Genes

**DOI:** 10.1371/journal.pone.0087869

**Published:** 2014-02-21

**Authors:** Mi-Rae Shin, Masaya Natsuume, Takashi Matsumoto, Mitsumasa Hanaoka, Misaki Imai, Ken Iijima, Shin-ichiro Oka, Eri Adachi, Hiroaki Kodama

**Affiliations:** 1 Graduate School of Advanced Integration Science, Chiba University, Inage-ku, Chiba, Japan; 2 Graduate School of Horticulture, Chiba University, Chiba, Japan; 3 Genome Research Center, NODAI Research Institute, Tokyo University of Agriculture, Setagaya-ku, Tokyo, Japan; 4 Department of Applied Biology and Chemistry, Tokyo University of Agriculture, Setagaya-ku, Tokyo, Japan; 5 Graduate School of Science and Technology, Chiba University, Inage-ku, Chiba, Japan; Tel Aviv University, Israel

## Abstract

Sense transgene-induced post-transcriptional gene silencing (S-PTGS) is thought to be a type of RNA silencing in which ARGONAUTE1 directs the small interfering RNA (siRNA)-mediated cleavage of a target mRNA in the cytoplasm. Here, we report that the altered splicing of endogenous counterpart genes is a main cause for the reduction of their mature mRNA levels. After the S-PTGS of a tobacco endoplasmic reticulum ω-3 fatty acid desaturase (*NtFAD3*) gene, 3′-truncated, polyadenylated *endo-NtFAD3* transcripts and 5′-truncated, intron-containing *endo-NtFAD3* transcripts were detected in the total RNA fraction. Although transcription proceeded until the last exon of the endogenous *NtFAD3* gene, intron-containing *NtFAD3* transcripts accumulated in the nucleus of the S-PTGS plants. Several intron-containing *NtFAD3* transcripts harboring most of the exon sequences were generated when an endogenous silencing suppressor gene, *rgs-CaM*, was overexpressed in the S-PTGS plants. These intron-containing *NtFAD3* splice variants were generated in the presence of *NtFAD3* siRNAs that are homologous to the nucleotide sequences of these splice variants. The results of this study indicate that the inhibition of *endo-NtFAD3* gene expression is primarily directed via the alteration of splicing and not by cytoplasmic slicer activity. Our results suggest that the transgene and intron-containing endogenous counterpart genes are differentially suppressed in S-PTGS plants.

## Introduction

In higher plants, a transgene often induces the inactivation of itself and homologous endogenous counterpart genes [1], [2], a phenomenon called sense transgene-induced post-transcriptional gene silencing (S-PTGS). In S-PTGS, aberrant transcripts from transgene loci recruit RNA-DEPENDENT RNA POLYMERASE 6 (RDR6) for the synthesis of complementary RNA strands [3], [4]. The resulting double-stranded RNAs (dsRNAs) are processed into 21 to 24-nucleotide (nt)-long small interfering RNAs (siRNAs) by DICER-LIKE proteins (DCLs) [5]. Single-stranded siRNAs are then incorporated into ARGONAUTE proteins (AGOs); in particular, siRNAs incorporated into AGO1 (also called slicer) act as a guide for mRNA degradation and/or translational inhibition [6], [7]. This silencing step is likely to occur at unique cytoplasmic foci, termed processing bodies (PBs), where AGO1, DCP2, and XRN4 are localized [8]. DCP2 is a decapping enzyme and XRN4 is an exoribonuclease; the impairment of the function of DCP2 and XRN4 induces RDR6-dependent S-PTGS [9], [10]. In the nucleus, the above-mentioned 24-nt-long siRNAs guide the methylation of homologous genomic DNA in a pathway referred to as RNA-directed DNA methylation (RdDM) [11], [12].

The degradation efficiency of the mRNAs originating from both a transgene and a homologous endogenous gene should be indistinguishable in S-PTGS if the target transcripts are primarily degraded by the cytoplasmic slicer complex. However, the transcript levels of the endogenous target genes are often more strongly reduced compared to the levels of the transgene transcripts. The introduction of a truncated *polygalacturonase* (*PG*) transgene into tomato plants causes S-PTGS during the ripening of tomato fruits, resulting in the accumulation of *PG* siRNA [13]. When this silencing-induced transgene locus was introduced into a tomato variety with a low level of endogenous *PG* gene expression during the ripening period, preferential suppression was observed for the endogenous *PG* gene and not the *PG* transgene [14]. In petunia plants, the introduction of the *CHALCONE SYNTHASE A* (*CHSA*) genes frequently resulted in S-PTGS [15]. Similar to the case of tomato *PG* S-PTGS, the loss of the endogenous *CHSA* transcripts in the S-PTGS lines was greater than the loss of transgene *CHSA* transcripts [16], and the *CHSA* S-PTGS was associated with the generation of *CHSA* siRNAs [17]. These results suggest a silencing pathway that preferentially suppresses endogenous target genes; however, no reports thus far have demonstrated such a silencing mechanism.

We previously investigated the S-PTGS of a gene (*NtFAD3*) encoding tobacco endoplasmic reticulum ω-3 fatty acid desaturase [18], an enzyme that catalyzes the conversion of linoleic acid (18∶2) to α-linolenic acid (18∶3) in membrane phospholipids. When the sense transgene (*trans-NtFAD3*) was introduced into tobacco plants, most of the resulting transgenic lines, such as the S20 and S24 lines, showed increased 18∶3 contents. In contrast, the S44 line showed a moderately reduced 18∶3 content in hemizygous plants and a severely reduced 18∶3 content in homozygous plants [19]. We also found that a high transcription rate of the transgene was necessary for the generation of *NtFAD3* siRNAs; when the promoter of the *trans-NtFAD3* gene was inactivated by RdDM, the transcription rate of the transgene was reduced, followed by the disappearance of *NtFAD3* siRNA. Because RdDM only partially inactivated the *trans-NtFAD3* transgene, its residual expression resulted in an increased leaf 18∶3 content compared to that of the wild-type (WT) leaves [20]. In the present study, we investigated the expression of the endogenous *NtFAD3* gene (*endo-NtFAD3*) in detail. Our results indicate that the S44 plants were compromised in the splicing of *endo-NtFAD3* pre-mRNA. In addition, the overexpression of a tobacco calmodulin-related protein that had been identified as an endogenous silencing suppressor [21] induced the accumulation of intron-containing *endo-NtFAD3* transcripts. These results indicate that the expression of the *endo-NtFAD3* gene was inhibited via the alteration of splicing steps in the nucleus.

## Results

### Detection of 3′- and 5′-truncated *endo-NtFAD3* RNAs

**Figure 1:**
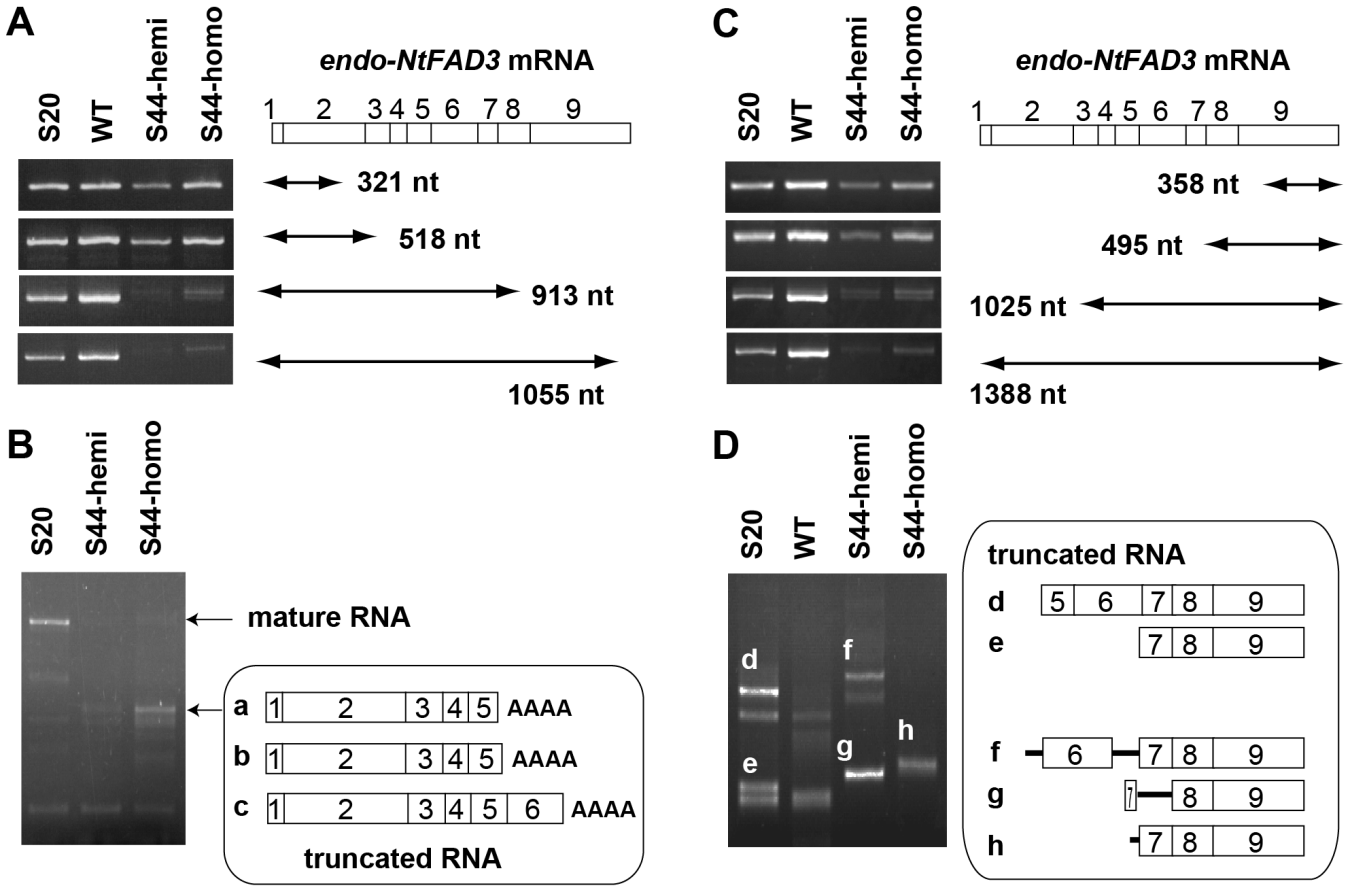
Detection of the 3′- and 5′-truncated *endo-NtFAD3* transcripts. (**A**) RT-PCR analysis of the *endo-NtFAD3* transcripts harboring the proximal region. S44-hemi and S44-homo denote the S44 plants hemizygous and homozygous for T-DNA, respectively. The amplified regions are illustrated. The *endo-NtFAD3* mRNA is shown with open boxes. Each box with a number shows the corresponding exon. (**B**) 3′ RACE analysis. The total RNAs from the S20, S44-hemi, and S44-homo leaves were subjected to 3′ RACE. The RACE products from mature and truncated *endo-NtFAD3* transcripts are indicated. The 3′ RACE products from truncated *endo-NtFAD3* transcripts were cloned as follows: ten independent clones were sequenced and classified into 3 groups (a, b, and c) based on the positions of their polyadenylation sites. (**C**) RT-PCR analysis of the *endo-NtFAD3* transcripts harboring the distal region. The amplified region is also illustrated, as in the case of Fig. 1A. (**D**) 5′ RACE analysis. The leaf total RNA was subjected to 5′ RACE. Five cDNA fragments (designated d to h) specific to the samples from the *NtFAD3* transformants were cloned, and the structures of the 5′-truncated *endo-NtFAD3* transcripts are shown. The open boxes and bars indicate exons and introns, respectively.

*Nicotiana tabacum* is an allotetraploid, and two distinct genes encoding the endoplasmic reticulum ω-3 fatty acid desaturase have been identified; *NtFAD3-1* (GenBank acc. no. AB049576) and *NtFAD3-2* (AB893595). The *NtFAD3* cDNA used in the construction of the transgene originated from the transcripts of the *NtFAD3-1* gene. Therefore, we focused on the expression of *NtFAD3-1*. Indeed, all of the *NtFAD3* sequences in this report were identified as *NtFAD3-1* sequences. Then the endogenous *NtFAD3-1* gene is simply indicated as *endo-NtFAD3*. The level of *endo-NtFAD3* mRNA was evaluated by RT-PCR analyses. The *NtFAD3* sense construct does not contain most of the 5′ untranslated sequence of the *endo-NtFAD3* gene, which is replaced by the Ω sequence [22]. Thus, the *endo-NtFAD3* mRNA can be distinguished from the *trans-NtFAD3* mRNA by RT-PCR analysis using primers corresponding to the 5′ untranslated region of the *endo-NtFAD3* gene. We primed cDNA synthesis using total leaf RNA with primers corresponding to exons 2, 4, 8, and 9 of the *NtFAD3* gene and then amplified the *endo-NtFAD3* cDNA. The length of the *NtFAD3* cDNA (GenBank acc. no. D26509) is 1366 bp, and we designated the upstream half region as proximal sequences and downstream half region as distal sequences. The proximal 321-bp- and 518-bp-long RT-PCR products were detectable in the total RNA from S44 leaves but the nearly full-length transcripts (913 bp and 1055 bp in length) were not (**Fig. 1A**), indicating that the accumulation of mature *endo-NtFAD3* mRNAs was severely suppressed in these leaves. As the results in **Fig. 1A** suggest the presence of *endo-NtFAD3* mRNA variants consisting of 5′ proximal exons, a rapid amplification of cDNA ends (RACE) was performed to identify the 3′ ends. The expected 1.4-kb product was detected in the sample from the overexpressing line S20 (indicated as mature RNA in **Fig. 1B**); conversely, this 1.4-kb product was absent in the S44 samples, although other major amplified products were detected (**Fig. 1B**). These short 3′ RACE products were classified into 3 groups based on the position of their 3′ ends (a, b, and c in **Fig. 1B**) and were polyadenylated at the internal sites of exons 5 and 6 (**Fig. S1**). Taken together, at least a portion of the *endo-NtFAD3* transcripts should be subjected to aberrant 3′ end processing in the leaves of the S44 plants.

Next, we investigated whether 5′-truncated *endo-NtFAD3* transcripts were generated in the S44 plants. We found an *endo-NtFAD3* transcript variant that had a 21-nt-long, extended 3′ untranslated region, and a primer corresponding to this extended sequence allowed us to specifically detect the distal region of the *endo-NtFAD3* mRNA. The *endo-NtFAD3* RT-PCR products (1025-bp- and 1388-bp-long) were clearly detected in the total RNA from the S20 and WT leaves but were barely detected in the S44 leaf total RNA (**Fig. 1C**). Therefore, the effects of S-PTGS were evident in the population of *endo-NtFAD3* mRNA variants harboring an extended 3′ untranslated region. Interestingly, the distal 358-nt and 495-nt fragments of the *endo-NtFAD3* mRNA were present in the S44 leaves, suggesting the existence of 5′-truncated *endo-NtFAD3* mRNA species (**Fig. 1C**). The 5′ termini of these mRNA species were then determined by 5′ RACE (**Fig. 1D**). Five major fragments distinct from any 5′ RACE products of the WT plants were obtained from the transgenic plants, designated d to h in **Fig. 1D**. The 5′ ends of the 5′ RACE products from S20 mRNA (d and e) were situated in the internal regions of exons 5 and 7, respectively; these two clones had no intronic sequences. In contrast, the 5′ RACE products from the S44 leaves (f, g, and h) retained intronic sequences (**Fig. 1D**). The 5′ cDNA ends of products f and h were located in introns 5 and 6, respectively; product g had an intron 7 sequence, and its 5′ end was mapped to the internal region of exon 6 (**Fig. S2**). These results indicate that, compared to the S20 and WT leaves, the *endo-NtFAD3* transcripts in the S44 silenced leaves are more abundantly present as 5′-truncated, aberrantly spliced forms.

**Figure 2:**
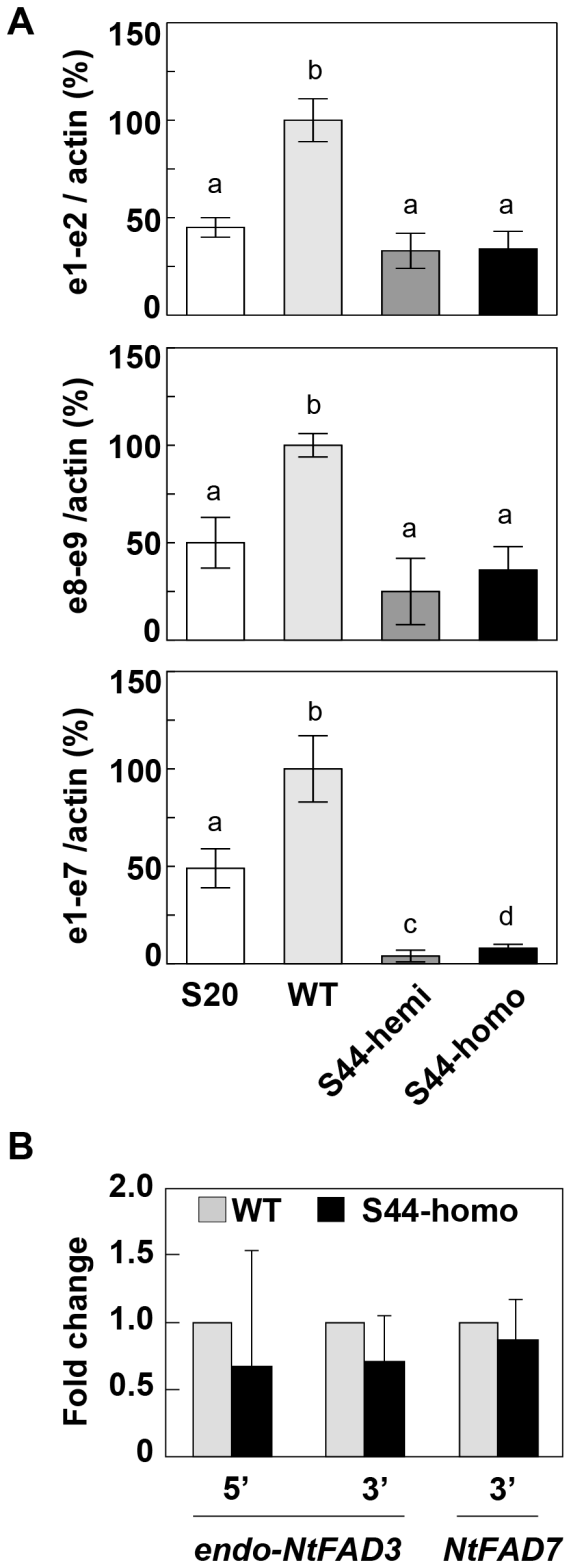
Expression levels of the *endo-NtFAD3* gene. (**A**) The transcript levels of the *endo-NtFAD3* gene in the total RNA fraction. Each *endo-NtFAD3* transcript level was determined by qRT-PCR and normalized to the level of actin cDNA. The normalized value for the amount of *endo-NtFAD3* mRNAs of the WT plants was considered to be 100%, and other normalized values in the S20, S44-hemi, and S44-homo plants were calculated as a percentage of that of the WT plants. e1-e2 and e8-e9 indicate the detection of *endo-NtFAD3* transcripts that contain the proximal region (exon 1 to exon 2) and distal region (exon 8 to exon 9), respectively. e1-e7 indicates the detection of *endo-NtFAD3* transcripts that contain a region from exon 1 to exon 7. The values are the mean ± SD (n = 3). (**B**) Pol II occupancy in the WT and S44 plants. ChIP-qPCR experiments were performed using an anti-Pol II antibody to detect the binding level at the indicated locus in the WT and the S44 plants. The mean of qPCR of the S44 sample is reported relative to the EF-1α gene control and is shown as a relative value of the corresponding WT level shown as 1.0. The graphical representation shows the fold change as the mean of the three different biological (and two different technical) replicates. The error bars represent SD. Different letters on the graph represent significantly different means (*P*<0.05).

The qRT-PCR analysis revealed that the *endo-NtFAD3* transcripts harboring exon 1 to exon 7 sequences were less abundantly generated in the S44 compared to the WT plants. Such a decrease in the *endo-NtFAD3* mRNA level was attenuated when the proximal region (exon 1 to exon 2) and the distal region (exon 8 to exon 9) of the *endo-NtFAD3* cDNA fragments were amplified using the S44 total RNA (**Fig. 2A**). These proximal and distal fragments should be amplified from cDNAs originating from 3′-truncated (**Fig. 1B**) and 5′-truncated *endo-NtFAD3* mRNA (**Fig. 1D**), respectively. On the other hand, the S20 line produced several truncated *endo-NtFAD3* transcripts (**Fig. 1D**). The qRT-PCR analysis showed a decreased level of the *endo-NtFAD3* mRNA in the S20 plants compared with that of the WT plants. Because these *NtFAD3*-overexpressed plants have no detectable *NtFAD3* siRNAs in a Northern analysis [19], the reduction of the *endo-NtFAD3* mRNA in the S20 plants is apparently independent of the siRNA-mediated RNA silencing. This inhibitory mechanism of the *endo-NtFAD3* gene remains to be clarified.

The RNA polymerase II (Pol II) occupancy at both the 5′ and 3′ regions of the *endo-NtFAD3* gene was not significantly different in the S44 and WT leaves (**Fig. 2B**). As a control, we investigated the Pol II occupancy at the 3′ end of the *NtFAD7* gene, which encodes a plastid ω-3 fatty acid desaturase, and found that the Pol II occupancy at this genomic sequence was nearly the same for the S44 and WT plants. These results indicate that transcript elongation in the S44 leaves proceeded to the 3′ terminal region of the *endo-NtFAD3* gene independently of the generation of aberrantly spliced transcripts.

### Profiling *NtFAD3* siRNAs

**Figure 3:**
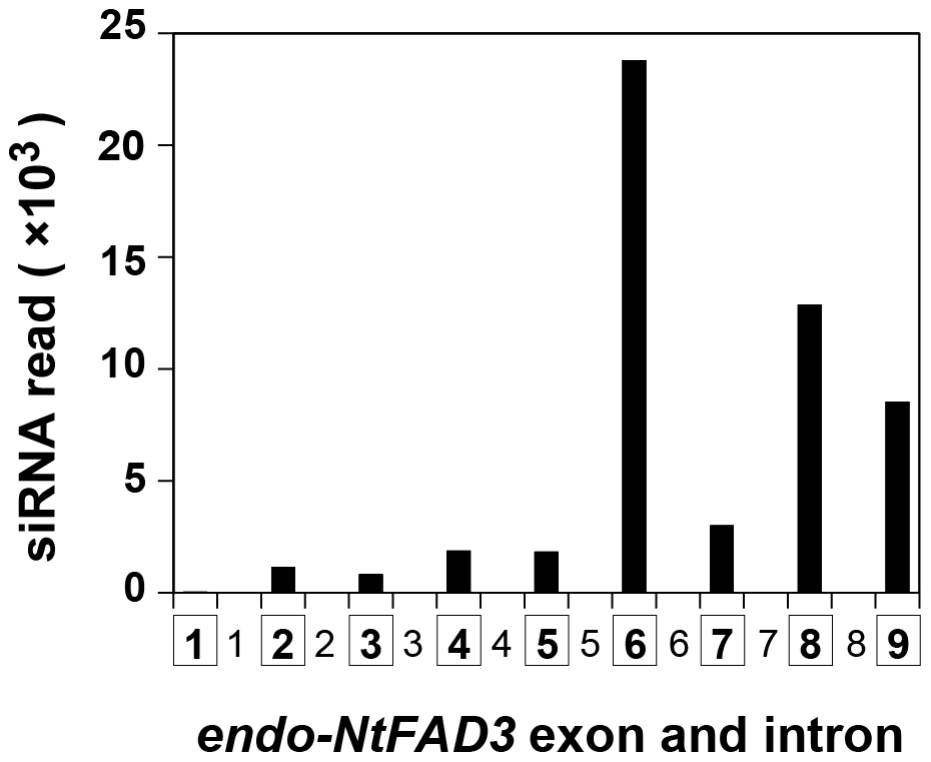
siRNA distribution along the *NtFAD3* gene. The read numbers of siRNAs mapped to each exon or intron are shown. The number in the squares indicates the exon numbers, and the other number corresponds to the intron number. Each siRNA was classified by its 5′ terminal nucleotide position relative to the *NtFAD3* sequence.

The key substance of RNA silencing is the siRNA molecule. We prepared small RNA libraries from the S44 and WT leaves. The deep sequencing of the WT small RNA library produced approximately 2.4×10^7^ sequence reads; only two 22-nt *NtFAD3* siRNAs were found, and these siRNAs harbored the identical sequence that was mapped to the sense strand of the sequence of *NtFAD3* exon 2. In contrast, the sequencing of the S44 small RNA library produced approximately 5.2×10^6^ sequence reads in total, and 53,312 sequence reads were mapped to the *NtFAD3* gene sequences. The *NtFAD3* siRNA population was dominated by species 22-nt (31%), 21-nt (16%), 23-nt (9%) and 24-nt (8%) in length; the remaining 36% of the *NtFAD3* siRNAs had lengths between 18 and 27 nt. When the siRNAs were mapped along the entire T-DNA region, most localized to the *NtFAD3* cDNA region (**Fig. S3**). Because a high transcription rate of the transgene was essential for generation of the *NtFAD3* siRNAs [20], most of the *NtFAD3* siRNAs were likely to be mainly generated from the *trans-NtFAD3* transcripts. To clarify the relationship between the altered splicing pattern of the *endo-NtFAD3* transcripts (**Fig. 1**) and the distribution of the *NtFAD3* siRNAs, the siRNAs were mapped to the *NtFAD3* genomic sequence. Approximately 90% of them localized to the regions of exons 6, 7, 8, and 9. The remaining 10% of the siRNAs mapped to exons 2, 3, 4, and 5 of the *NtFAD3* gene (**Fig. 3**). The antisense *NtFAD3* siRNAs that had been mapped to the exon 6 region were extremely abundant (**Fig. S3**), indicating the presence of hot spots for accumulation of siRNAs. The localized and uneven production of the siRNAs was observed in the S-PTGS of the petunia *CHSA* gene [23], [24]. At present, a mechanism of the generation of hot spot in the siRNA distribution is not clear. We found two siRNAs harboring intron 1 and intron 6 sequences of the *NtFAD3* gene, and these two siRNAs were very rare in the siRNA population because the read number of each siRNA species was only one. This result indicates that aberrantly spliced, intron-containing *endo-NtFAD3* transcripts (**Fig. 1D**) rarely serve as a template of RDR6.

We then compared the terminal nucleotides of the aberrantly spliced products (**Figs. 1B and 1D**) with the possible cleavage sites of *NtFAD3* siRNAs. We found no antisense-stranded *NtFAD3* siRNA that could potentially produce the 3′-truncated or 5′-truncated *NtFAD3* mRNAs if siRNA guides mRNA cleavage at the middle position of the siRNA strand. This result suggest that the aberrantly spliced products are not the direct products of pre-mRNA cleavage guided by siRNAs.

### Accumulation of Intron-containing *endo-NtFAD3* Transcripts in the S44 Nuclei

**Figure 4:**
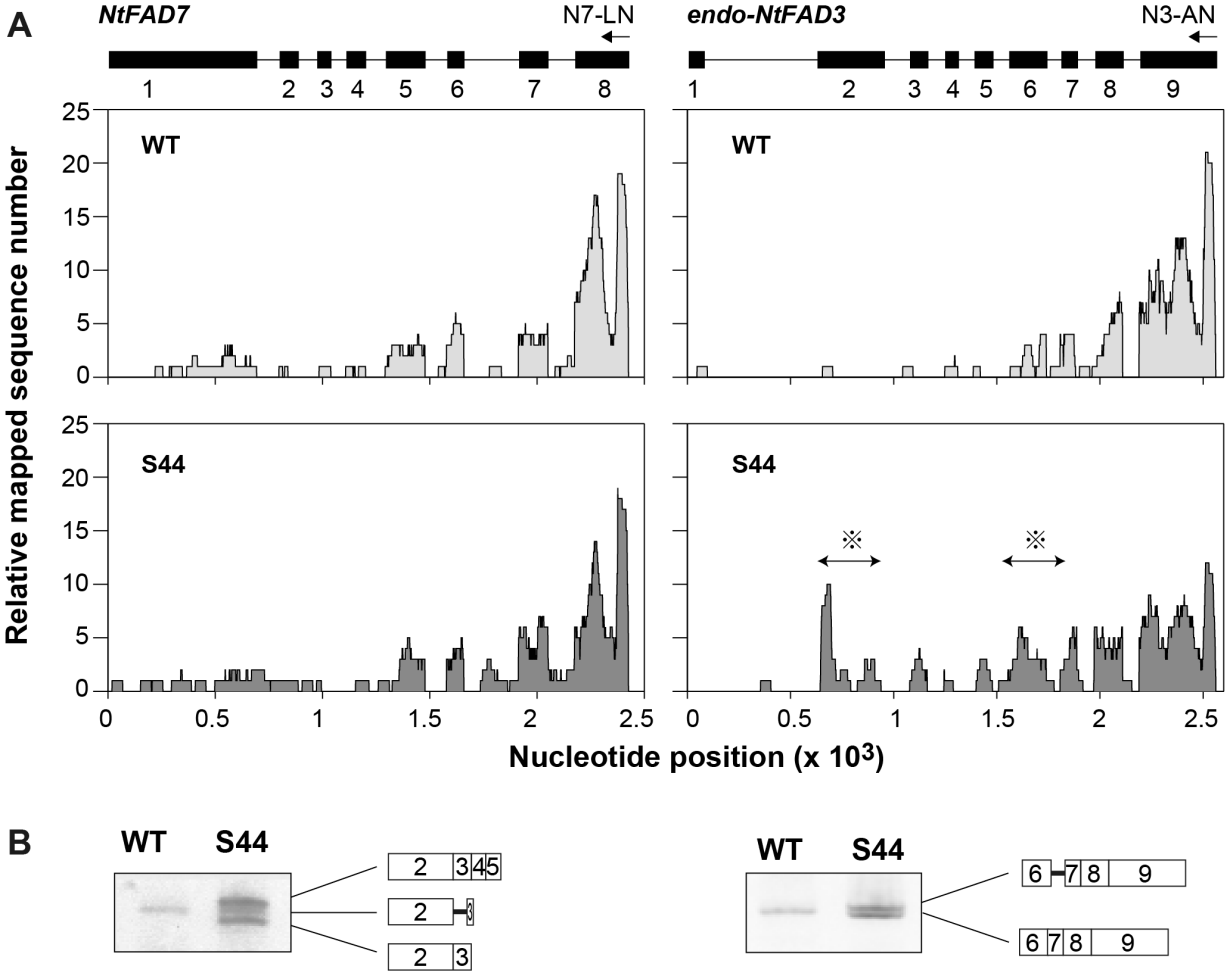
The *endo-NtFAD3* transcripts in the nuclei. (**A**) Nuclear RNA was reverse transcribed with gene-specific primers (N7-LN for the *NtFAD7* gene and N3-AN for the *endo-NtFAD3* gene) and then converted into double-stranded cDNA. The cDNAs were analyzed by Illumina sequencing. The read sequences were mapped to the genomic sequences, and the relative distribution of the mapped read sequences is shown. The genomic structure of the *NtFAD7* and *NtFAD3* genes are shown at the top of the panel on the same x-axis scale of the corresponding graphs. The asterisks show the unusual distribution of the read sequences along the exon 2 and exon 6 sequences of the *endo-NtFAD3* gene. (**B**) Detection of intron-containing nuclear transcripts. Nuclear transcripts harboring exon 2 sequences were amplified with the N7-LN and exon 2-specific forward primers. Three types of transcripts were cloned, and their structures are illustrated. Nuclear transcripts harboring exon 6 sequences were amplified with the N3-AN and exon 6-specific forward primers. The exons are illustrated with open boxes; the introns are shown with solid bars.

To reveal the structure of the nascent *endo-NtFAD3* transcripts, nuclear RNA was prepared and then reverse-transcribed by AMV reverse transcriptase (RTase) with three gene-specific primers, N3-AN, N7-LN, and EF-1α-Rv, corresponding to the 3′ distal sequences of the *endo-NtFAD3*, *NtFAD7*, and EF1α genes, respectively. The resulting cDNAs were deep-sequenced using the Illumina system. As the elongation capacity of AMV RTase decreases when AMV RTase synthesizes a cDNA strand of 1 kb or more, many of the read sequences were mapped to the 3′ distal region of the target gene, with very few sequences mapping to the 5′ proximal region. This typical distribution of mapped read sequences was observed with the *NtFAD7* transcripts from both the S44 and WT nuclei (**Fig. 4A**). A similar distribution of read sequences was evident when the *endo-NtFAD3* transcripts in the WT nuclei were analyzed; however, the distribution of *NtFAD3* cDNA reads in the S44 samples was significantly different (**Fig. 4A**). The read numbers of the sequences harboring exon 2 or exon 6 sequences from the *NtFAD3* gene were significantly higher in the S44 plants than those in the WT plants (shown by asterisks in **Fig. 4A**; *P*<0.01). To determine these unique nascent RNA species found in the S44 plants, several RT-PCR analyses were performed. The intron 6-containing *NtFAD3* transcripts were detected by RT-PCR with the N3-AN and exon 6-specific forward primers. In contrast, an intron 2-containing *NtFAD3* transcript and two transcripts harboring proximal exonic *NtFAD3* sequences were detected by RT-PCR using an *NtFAD7* reverse primer (N7-LN) and exon 2-specific primer (Exon2-fw2) (**Fig. 4B**). The cDNAs containing the *NtFAD3* exon 2 sequence were likely generated by reverse transcription with the N7-LN primer, which showed a partial sequence similarity to the *NtFAD3* sequences. We designed a reverse primer (Ex3-N7-LN) from the *NtFAD3* sequence that had been expected to be annealed with the N7-LN primer. Interestingly, the RT-PCR with a primer pair, Ex3-N7-LN/Exon2-fw2 produced a different electrophoretogram from that of the RT-PCR with a primer pair, N7-LN/Exon2-fw2 (**Fig. S4**). Both an intron 2-containing fragment and a fragment consisting of exonic sequences were amplified from both the WT and S44 nuclear RNAs when a primer pair, Ex3-N7-LN/Exon2-fw2, was used. It is possible that the *NtFAD3* transcripts detected with the N7-LN primer (**Fig. 4B**) contain modified sequences at their 3′ terminal region and the N7-LN primer anneals with these modified sequences. However, at present we have no additional data about the structures of these nascent *NtFAD3* transcripts detected with the N7-LN primer. Taken together, these results indicate that the proximal fragments of the *NtFAD3* transcripts harboring exon 2- and intron 6-retaining transcripts accumulated in the S44 nuclei.

### Enhanced Accumulation of Intron-containing *NtFAD3* Transcripts by the Ectopic Expression of a Calmodulin-related Gene

**Figure 5:**
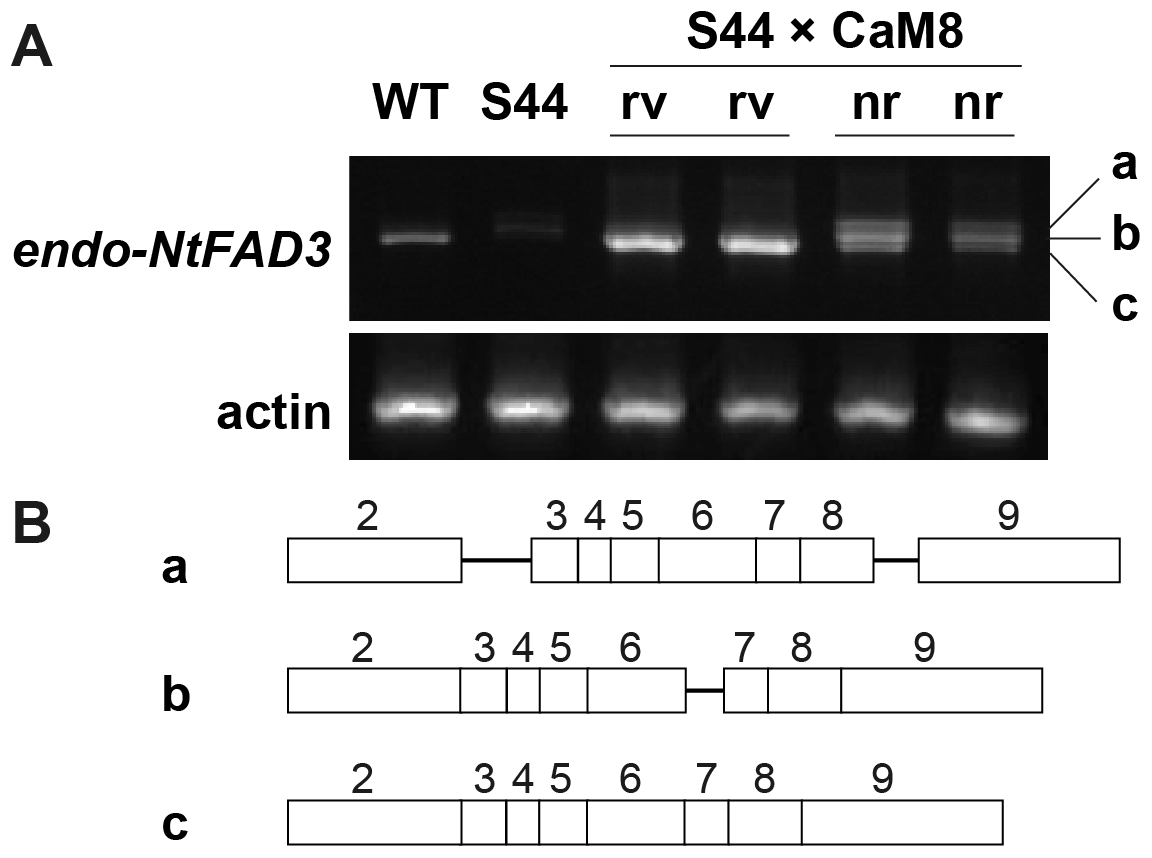
Effects of the ectopic expression of the *rgs-CaM* gene on *endo-NtFAD3* transcripts. (**A**) RT-PCR analysis of *endo-NtFAD3* transcripts. The levels of *endo-NtFAD3* transcripts were determined by RT-PCR analysis using the exon 2-specific and N3-AN primers. The equivalence of the amount of RNA used in RT-PCR is shown by the amplification of actin cDNA fragments. Two independent revertants (rv) and two independent non-revertants (nr) were identified among the descendants of a crossbred line (S44×CaM8) by measuring the leaf 18∶3 contents [23]. Three cDNA fragments found in the non-revertant lines were cloned and sequenced. (**B**) Structures of the *endo-NtFAD3* transcripts generated in the non-revertant plants. The exons and introns are shown with open boxes and solid bars. The exon numbers are shown on the corresponding boxes.

A tobacco calmodulin-related protein, rgs-CaM, modulates host RNA silencing [21]. When the *rgs-CaM* gene was ectopically overexpressed in the S44 plants, 70% of the progeny showed a large decrease in the level of *NtFAD3* siRNA and the successful overexpression of the *trans-NtFAD3* gene. Here, we call these plants ‘revertants’. The remaining 30% of the descendants generated the *NtFAD3* siRNAs and showed a low 18∶3 phenotype in leaves; we designate these S-PTGS plants as ‘non-revertants’ [25]. When the *endo-NtFAD3* cDNA corresponding to the exon 2 to exon 9 regions was amplified using the total RNA fraction, cDNA fragments consisting of exon sequences were detected in the WT and revertant plants but rarely found in the S44 plants. In contrast, three cDNA fragments were clearly amplified in the non-revertant plants (**Fig. 5A**). The most abundantly amplified cDNA harbored sequences of intron 6, and the largest cDNA fragment contained intron 2 and intron 8 sequences (**Fig. 5B**). These results indicate that rgs-CaM induces the accumulation of intron-containing *endo-NtFAD3* transcripts in the non-revertant plants. The rgs-CaM protein binds to the dsRNA-binding domain of the 2b and HC-Pro proteins [26]. Although the function of rgs-CaM in the nucleus is unknown, it is possible that rgs-CaM interacts with the nuclear RNA silencing machinery and then alters the splicing of the *endo-NtFAD3* transcripts in the non-revertant plants.

## Discussion

The RNA silencing pathway, particularly the cytoplasmic slicer-mediated mRNA cleavage pathway, has long been considered a primary cause of the knockdown of endogenous target genes. One reason for this hypothesis is the suppression of viral amplification by RNA silencing [27], [28]. RNA viruses replicate exclusively in the cytoplasm [29], [30], and the suppression of viral replication by RNA silencing should be associated with the cytoplasmic RNA cleavage activity of the siRNA-AGO1 protein complex [6]. However, this silencing pathway cannot explain the preferential knockdown of endogenous target genes that is observed in several S-PTGS plants. The preferential suppression of endogenous target genes is often associated with the generation of aberrantly spliced products, such as truncated *CHSA* mRNAs [16], truncated β-1,3-glucanase mRNAs [31], and intron-containing β-1,3-glucanase mRNAs [32]. Mishra and Handa [33] also reported that a sense transgene for the pectin methylesterase gene (*PME*) induced the accumulation of unspliced endogenous *PME* transcripts. These early studies of S-PTGS often proposed that nuclear events, including splicing, would be the primary site of action of RNA silencing. However, in most cases, there has long been a consensus that cytoplasmic mRNA degradation is a main cause and that aberrant splicing is not an essential step for the RNA silencing of endogenous genes [32]. Nonetheless, our results suggest that cytoplasmic mRNA degradation is not a main cause of endogenous *NtFAD3* gene knockdown. *NtFAD3* siRNAs harboring exon 6, 7, 8, and 9 sequences were abundant in the S44 plants (**Fig. 3**), whereas *endo-NtFAD3* transcripts harboring exon 8 and 9 sequences accumulated to a comparable level in the S20 line (**Fig. 1D**), and ectopically expressed rgs-CaM induced the accumulation of *endo-NtFAD3* splice variants harboring exon 2 to 9 sequences in the non-revertant plants (**Fig. 5B**). Therefore, RNA degradation by *NtFAD3* siRNAs was not efficient in the S44 and non-revertant plants. In contrast, the splicing of nascent *endo-NtFAD3* transcripts was altered, and various splice variants were produced; this altered splicing must be the main cause of the reduction of mature mRNAs in the S44 plants. Interestingly, the dsRNAs targeting intronic sequences of the soybean ω-6 desaturase gene (*FAD2*) can silence *FAD2* expression, indicating that pre-mRNA is a target of RNA silencing [34].

The splice variants identified in the total RNA fraction (**Fig. 1**) have different structures than the splice variants observed in the nuclear RNA fraction (**Fig. 4**). Because splice variants with premature termination codons are preferentially degraded by nonsense-mediated mRNA decay (NMD) [35], it is possible that *NtFAD3* siRNAs are involved in the generation of various types of splice variants, though some of these variants are efficiently eliminated by NMD. Therefore, the splice variants that evade NMD are identified in the total RNA fraction (**Fig. 1** **,** **Fig. 5**) and account for only a small portion of the total variants generated in the S44 plants.

There is no information on the RNA silencing components that are involved in the modulation of splicing. Pol II and AGO4 may function together at some RdDM target sites, indicating that AGO4 can interact with Pol II transcripts [36]. However, a splicing alteration due to the interaction between siRNA and pre-mRNA has not been reported in higher plants. In mammalian cells, intronic siRNAs affects alternative splicing [37]. Further investigation is necessary to evaluate the mechanism of the siRNA-mediated alteration of splicing in the S-PTGS pathway.

## Materials and Methods

### Plant Materials

Primary transformed tobacco (*Nicotiana tabacum* cv SR1) plants (R_0_) introduced with a sense *NtFAD3* transgene were generated as previously described [19]. Crosses between the S44 plants and plants overexpressing the *rgs-CaM* gene (CaM8 line) were as previously described [25]. The seedlings of these transgenic plants were transferred to the soil, cultured in continuous light at 26°C, and subjected to further gene expression analyses.

### RT-PCR Analysis

Total RNA was isolated with TRIzol reagent (Life Technologies) according to the manufacturer’s protocols. When the endogenous *NtFAD3* transcripts were analyzed, 1 µg total RNA was reverse transcribed by AMV RTase (Promega). The primers used in the RT-PCR analyses are listed in **Table S1**. The electrophoretograms showed essentially the same patterns when the amplified products at 24, 27 and 30 cycles of the PCR reaction were analyzed to avoid the saturation of DNA amplification.

### 3′ and 5′ RACE

The 3′ RACE was performed using the TaKaRa RNA LA PCR Kit version 1.1 (TaKaRa). The first-strand cDNA was primed using total RNA as the template with an oligo-dT adapter primer. The 3′-terminal regions were amplified by PCR using an adapter primer and primers specific for the *endo-NtFAD3* mRNA (**Table S2**).

The 5′ RACE was performed using the 5′ RACE System version 2.0 (Gibco BRL). Using total RNA, the first-strand cDNA was primed with a primer specific to the *endo-NtFAD3* transcript (**Table S2**). These RACE products were cloned and sequenced.

### qRT-PCR Assay

cDNA was synthesized from 1 µg of total RNA and primers for the *endo-NtFAD3* and actin genes. Each PCR mixture contained 1/40 of the reverse transcription mixture and cDNAs were amplified with Tbr EXT DNA polymerase (Finnzymes). The primers used are listed in **Table S3**. The PCR amplification was performed using a Rotor-Gene (Corbett Research), and the amplified fragments were detected by staining with SYBR Green (Molecular Probe Inc.). A melting temperature profile and agarose gel analysis of the PCR products showed that non-specific amplification did not occur.

### Chromatin Immunoprecipitation (ChIP)-qPCR Assay

A ChIP-qPCR assay was performed as previously described [38], [39]. An anti-RNA polymerase II CTD repeat YSPTSPS antibody (ab817; Abcam) was used for the immunoprecipitation of Pol II-DNA complexes. The primers used for ChIP-qPCR are listed in **Table S3**.

### Isolation of Nuclei and Preparation of Nuclear RNA

Nuclei were isolated essentially according to the method of van Blokland et al. [40]. Fresh leaf tissues were ground in liquid nitrogen and the powder was suspended in ice-cold buffer A (10 mM NaCl, 10 mM MES (pH 6.0), 5 mM EDTA, 0.15 mM spermine, 0.5 mM spermidine, 5 mM 2-mercaptoethanol, 0.6% (v/v) Triton X-100 and 0.25 M sucrose). The suspension was passed through four layers of cheesecloth; the filtrate was layered onto buffer B (6 g of 5×buffer A and 45 g Percoll) and centrifuged for 10 min at 1500×*g*. Most of the nuclei banded just above the buffer B cushion. After the collection of these nuclei, the suspension was diluted by the addition of an equal volume of buffer A and centrifuged at 1500×*g* for 10 min. The nuclear pellet was washed once with buffer A, and the nuclei were resuspended in RNA extraction buffer (0.1 M Tris-HCl (pH9.0), 0.3 M NaCl, 10 mM EDTA, 0.1% sodium lauroyl sarcosinate, and 10 mM dithiothreitol). After the phenol/chloroform extraction, the total nucleic acids were precipitated with isopropanol. The nucleic acids were dissolved in 50 mM Tris-HCl (pH7.5) containing 5 mM MgCl_2_, and then RNase-free DNase I (TaKaRa) was added. The reaction mixture was incubated at 37°C for 30 min. The RNA was precipitated with ethanol after the phenol/chloroform extraction. The biologically different nuclear RNA samples were prepared for deep-sequencing (**Fig. 4A**) and RT-PCR analyses (**Fig. 4B**).

### Deep-sequencing Analysis of Small RNA

The PCR amplification of the small RNA library and the nucleotide sequence of the amplified cDNA analyzed using the Illumina Genome Analyzer (GAII) were performed by Hokkaido System Science Co. Ltd. The adapter sequence was trimmed from the raw short-read data, and the resulting short reads (18 to 31 nt) were mapped to the nucleotide sequence of the T-DNA region or the *NtFAD3* genomic region (GenBank acc. no. AB049576). The sequencing data were deposited in the DDBJ Sequence Read Archive (DRA) under accession number DRA001214.

### Deep-sequencing Analysis of Nuclear RNAs

The nuclear RNA was further purified using the RNeasy Mini Kit (Qiagen). Two independently prepared nuclear RNA samples were equally mixed and then reverse-transcribed by AMV RTase with the primers shown in **Table S4**. Double-stranded cDNA was prepared using a PCR-Select cDNA Subtraction Kit (Clontech) and then fragmented by ultrasonication. The cDNAs were Illumina-sequenced after size fractionation by agarose gel electrophoresis. The resulting 50-nt reads were mapped to the *NtFAD3* and *NtFAD7* (GenBank acc. no. AB049577) genomic sequences using the TopHat software with the default parameters [41]. The sequencing data were deposited in the DDBJ Sequence Read Archive (DRA) under accession number DRA001214.

## Supporting Information

Figure S1 **Terminal nucleotides of the 3′-truncated endo-NtFAD3 transcripts.** (**A**) The position of the terminal nucleotide of the 3′-truncated *endo-NtFAD3* transcripts. The RACE products were cloned and sequenced based on the 3′ RACE analysis shown in Fig. 1; the clones were classified into 3 groups according to the product length. The numbers indicate the 3′ terminal nucleotide positions that were mapped to the *endo-NtFAD3* genomic sequences. (**B**) The 3′ terminal sequences of the RACE products are shown in comparison to the *NtFAD3* cDNA sequence. AAAAn denotes a polyadenylated sequence.
(TIF)

Figure S2 **Terminal nucleotides of the 5′-truncated endo-NtFAD3 transcripts.** (**A**) The position of the terminal nucleotide of the 5′-truncated *endo-NtFAD3* transcripts. The RACE products were cloned and sequenced based on the 5′ RACE analysis shown in Fig. 1; the clones were classified into 3 groups according to the product length. The numbers indicate the 5′ terminal nucleotide positions that were mapped to the *endo-NtFAD3* genomic sequences. (**B**) The 5′ terminal sequences of the RACE products are shown in comparison to the *NtFAD3* genomic sequence. nGGGG is a tailed nucleotide synthesized for the amplification of the 5′ ends.
(TIF)

Figure S3 **Distribution of siRNAs along the T-DNA sequence.** The positive or negative y-axis shows the number of siRNAs mapped to the sense and antisense strands, respectively, with respect to the T-DNA sequence. NPT indicates the neomycin phosphotransferase II gene. Pel2Ω indicates the enhanced cauliflower mosaic virus promoter sequence. Tnos indicates the terminator sequence from the nopaline synthase gene. The asterisk shows the read number of the antisense-stranded siRNAs harboring *NtFAD3* exon 6 sequences.
(TIF)

Figure S4 **Analysis of the RT-PCR products with the N7-LN primer.** (**A**) Comparison of RT-PCR products amplified from the WT and S44 nuclear RNAs. The same figure was cited from Figure 4B. The fragment b was further analyzed. (**B**) Comparison of primer sequences with the partial exon 3 sequences of the *NtFAD3* cDNA. We cloned the fragment b, and the *NtFAD3* sequence that had been annealed with the N7-LN primer was deduced. Then a primer, Ex3-N7-LN, was designed. (**C**) Comparison of the RT-PCR products with the primer pairs of N7-LN/Exon2-fw2 and Ex3-N7-LN/Exon2-fw2. The nuclear RNAs were reverse transcribed with the N3-AN, N7-LN, and EF-1α-Rv primer mix, and then amplified using indicated primer pairs.
(TIF)

Table S1 **List of primers used in the RT-PCR analyses.**
(DOC)

Table S2 **List of primers used in the RACE analyses.**
(DOC)

Table S3 **List of primers used for the data in** **Figure 2** **.**
(DOC)

Table S4 **List of primers used for the deep sequencing of nuclear RNAs.**
(DOC)
