# Supplementary figures and images for: Sense Transgene-Induced Post-Transcriptional Gene Silencing in Tobacco Compromises the Splicing of Endogenous Counterpart Genes
Source: PLoS One. 2014 Feb 21;9(2):e87869. doi: 10.1371/journal.pone.0087869 (PMC3931610; doi:10.1371/journal.pone.0087869)

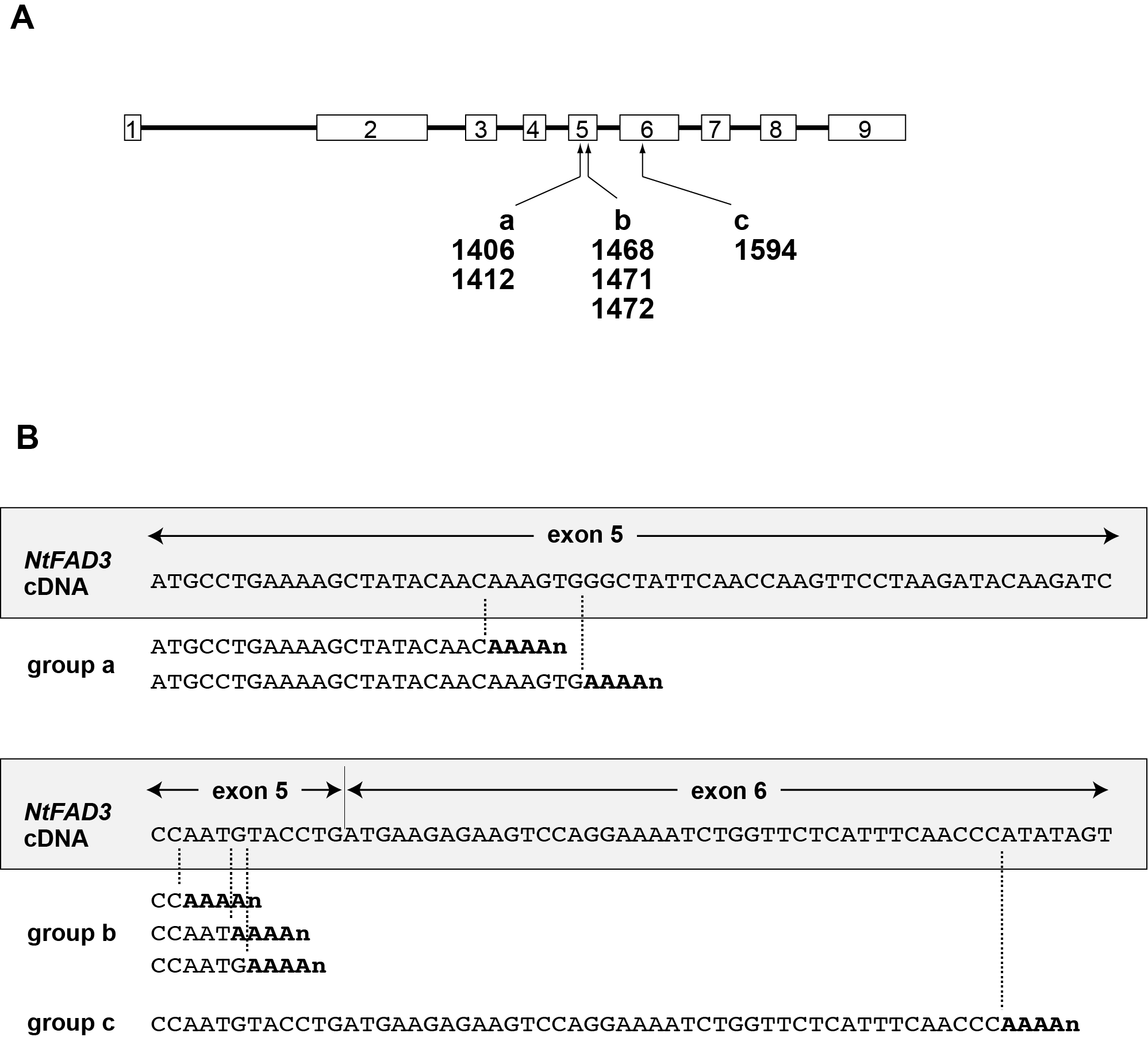

Supplement: Figure S1 — Terminal nucleotides of the 3′-truncated endo-NtFAD3 transcripts. (A) The position of the terminal nucleotide of the 3′-truncated endo-NtFAD3 transcripts. The RACE products were cloned and sequenced based on the 3′ RACE analysis shown in Fig. 1; the clones were classified into 3 groups according to the product length. The numbers indicate the 3′ terminal nucleotide positions that were mapped to the endo-NtFAD3 genomic sequences. (B) The 3′ terminal sequences of the RACE products are shown in comparison to the NtFAD3 cDNA sequence. AAAAn denotes a polyadenylated sequence. (TIF) [file pone.0087869.s001.tif]

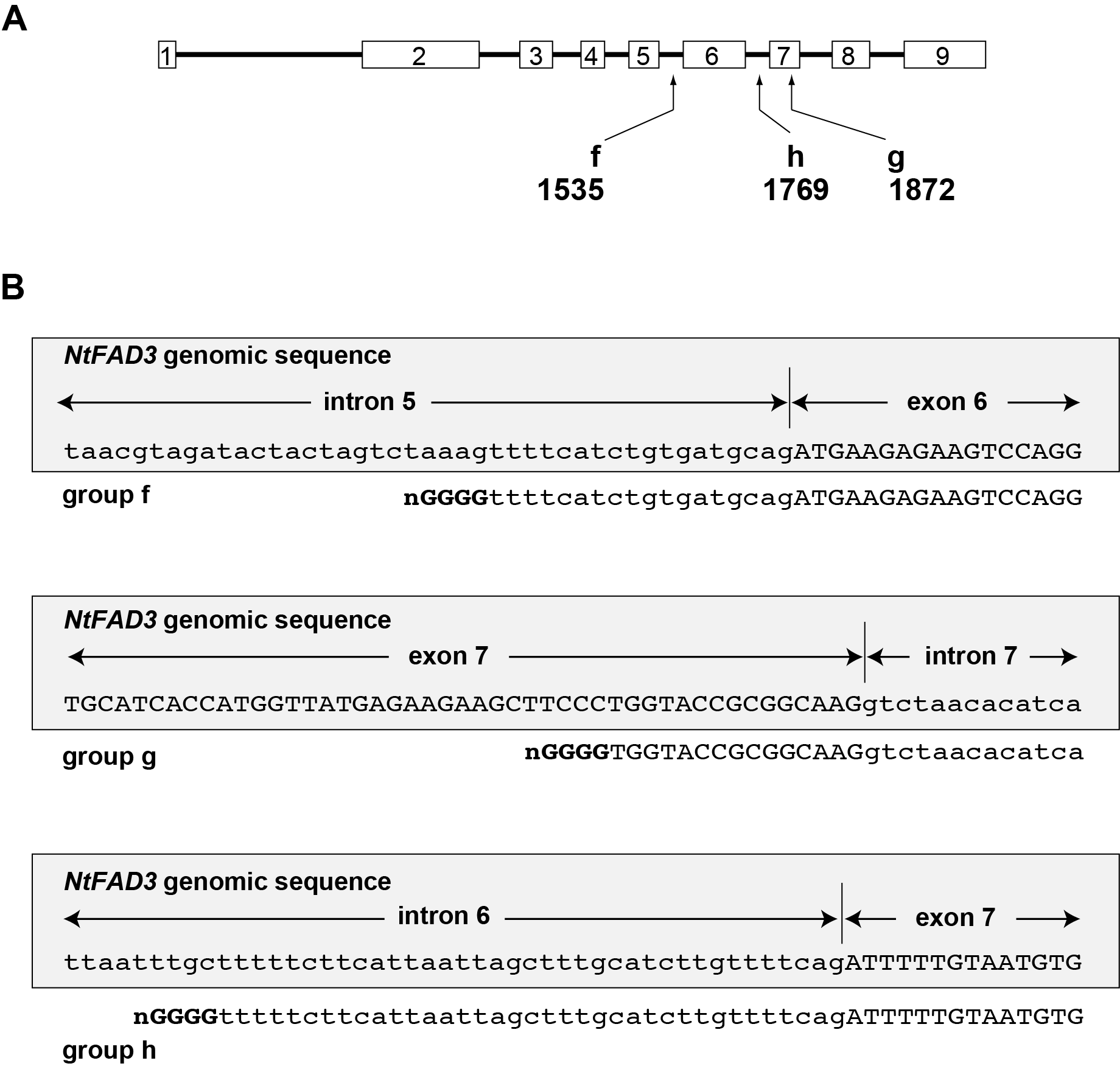

Supplement: Figure S2 — Terminal nucleotides of the 5′-truncated endo-NtFAD3 transcripts. (A) The position of the terminal nucleotide of the 5′-truncated endo-NtFAD3 transcripts. The RACE products were cloned and sequenced based on the 5′ RACE analysis shown in Fig. 1; the clones were classified into 3 groups according to the product length. The numbers indicate the 5′ terminal nucleotide positions that were mapped to the endo-NtFAD3 genomic sequences. (B) The 5′ terminal sequences of the RACE products are shown in comparison to the NtFAD3 genomic sequence. nGGGG is a tailed nucleotide synthesized for the amplification of the 5′ ends. (TIF) [file pone.0087869.s002.tif]

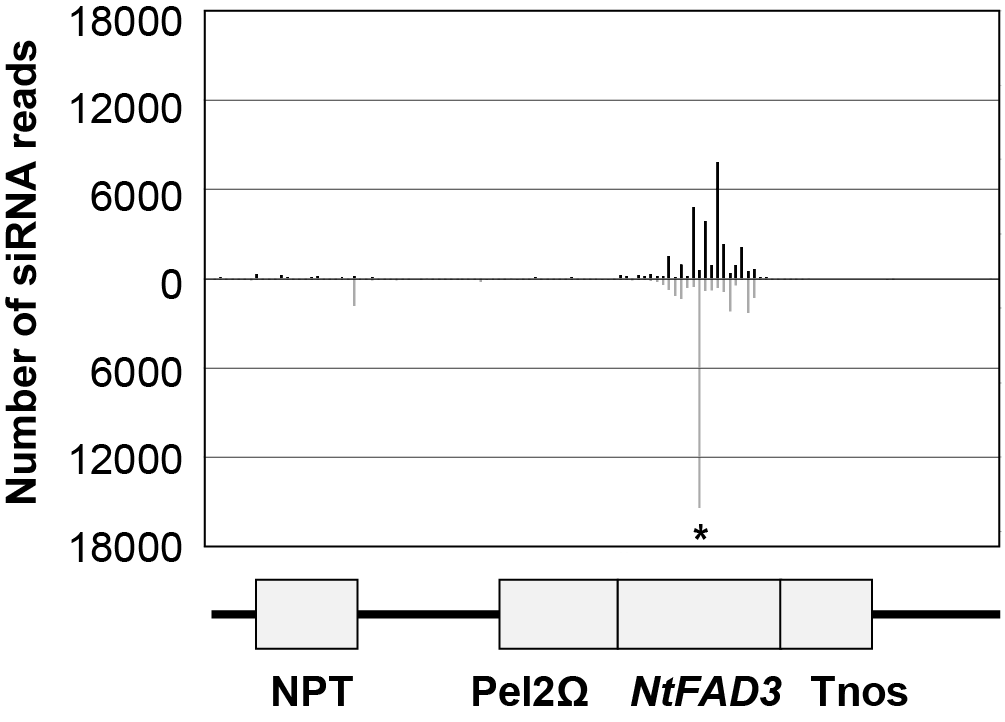

Supplement: Figure S3 — Distribution of siRNAs along the T-DNA sequence. The positive or negative y-axis shows the number of siRNAs mapped to the sense and antisense strands, respectively, with respect to the T-DNA sequence. NPT indicates the neomycin phosphotransferase II gene. Pel2Ω indicates the enhanced cauliflower mosaic virus promoter sequence. Tnos indicates the terminator sequence from the nopaline synthase gene. The asterisk shows the read number of the antisense-stranded siRNAs harboring NtFAD3 exon 6 sequences. (TIF) [file pone.0087869.s003.tif]

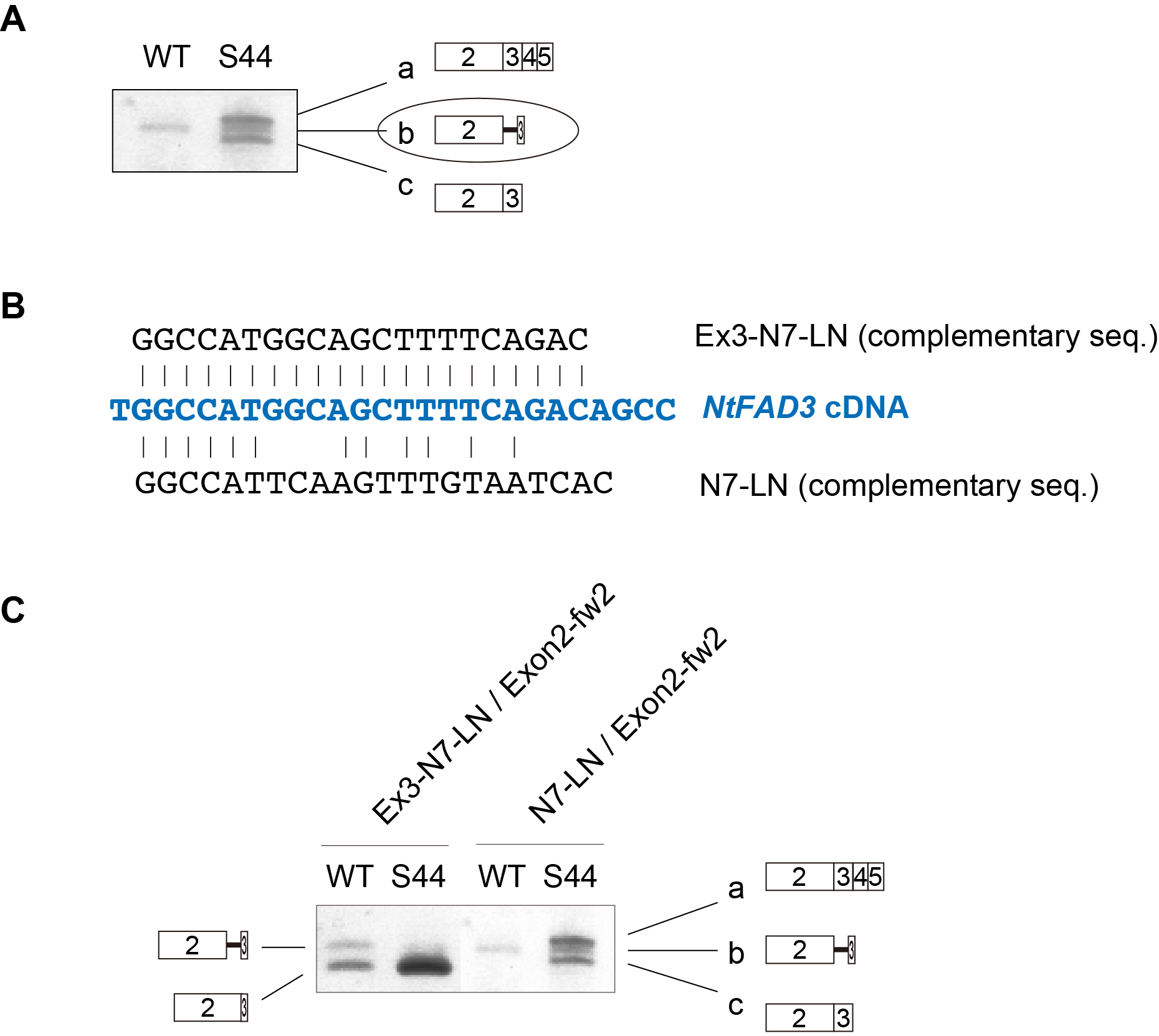

Supplement: Figure S4 — Analysis of the RT-PCR products with the N7-LN primer. (A) Comparison of RT-PCR products amplified from the WT and S44 nuclear RNAs. The same figure was cited from Figure 4B. The fragment b was further analyzed. (B) Comparison of primer sequences with the partial exon 3 sequences of the NtFAD3 cDNA. We cloned the fragment b, and the NtFAD3 sequence that had been annealed with the N7-LN primer was deduced. Then a primer, Ex3-N7-LN, was designed. (C) Comparison of the RT-PCR products with the primer pairs of N7-LN/Exon2-fw2 and Ex3-N7-LN/Exon2-fw2. The nuclear RNAs were reverse transcribed with the N3-AN, N7-LN, and EF-1α-Rv primer mix, and then amplified using indicated primer pairs. (TIF) [file pone.0087869.s004.tif]
