# Supplementary material for: Sense Transgene-Induced Post-Transcriptional Gene Silencing in Tobacco Compromises the Splicing of Endogenous Counterpart Genes
Source: PLoS One. 2014 Feb 21;9(2):e87869. doi: 10.1371/journal.pone.0087869 (PMC3931610; doi:10.1371/journal.pone.0087869)
Supplement: Table S1 — List of primers used in the RT-PCR analyses. (DOC) [file pone.0087869.s005.doc]

| Figure 1A |  |  |
| --- | --- | --- |
| cDNA length | primer | sequence (5’-3’) |
| 321 bp | N3-LC1 | GCAGTGTAGAGGAATACGGA |
|  | N3-N3 | CCTTGGATAGCCCAGTAAAG |
| 518 bp | N3-LC1 | GCAGTGTAGAGGAATACGGA |
|  | N3-N2 | TCGGCACCCAAGACTCATCA |
| 913 bp | N3-LC1 | GCAGTGTAGAGGAATACGGA |
|  | N3-Ne8 | AACTGTGGTTAGTCCTCCTC |
| 1055 bp | N3-LC1 | GCAGTGTAGAGGAATACGGA |
|  | N3-LN2 | CCCTGTAATACTTGCCAAGTACT |
| Figure 1C |  |  |
| cDNA length | primer | sequence (5’-3’) |
| 358 bp | N3-C2 | CCAGTACTTGGCAAGTATTA |
|  | N3-AN | ACTAAAGAAAGCCCTGTTCTTT |
| 495 bp | N3-Ce8 | GAGGAGGACTAACCACAGTT |
|  | N3-AN | ACTAAAGAAAGCCCTGTTCTTT |
| 1025 bp | N3-C1 | CCATGGCAGCTTTTCAGACA |
|  | N3-AN | ACTAAAGAAAGCCCTGTTCTTT |
| 1388 bp | N3-LC1 | GCAGTGTAGAGGAATACGGA |
|  | N3-AN | ACTAAAGAAAGCCCTGTTCTTT |
| Figure 4B and 4C |  |  |
| amplified region | primer | sequence (5’-3’) |
| exon 2 | Exon2-fw2 | GGGAATAAGTGAGATTTATGATAAG |
|  | N7-LN | GTGATTACAAACTTGAATGGCC |
| exon 6 | Exon6-fw2 | GTATGTGGTAACATCAACTCTGT |
|  | N3-AN | ACTAAAGAAAGCCCTGTTCTTT |
| Figure 5A |  |  |
| amplified region | primer | sequence (5’-3’) |
| exon 2 – exon 9 | Exon2-fw2 | GGGAATAAGTGAGATTTATGATAAG |
|  | N3-AN | ACTAAAGAAAGCCCTGTTCTTT |
|  |  |  |
