# Supplementary material for: Sense Transgene-Induced Post-Transcriptional Gene Silencing in Tobacco Compromises the Splicing of Endogenous Counterpart Genes
Source: PLoS One. 2014 Feb 21;9(2):e87869. doi: 10.1371/journal.pone.0087869 (PMC3931610; doi:10.1371/journal.pone.0087869)
Supplement: Table S2 — List of primers used in the RACE analyses. (DOC) [file pone.0087869.s006.doc]

| 3’ RACE |  |  |
| --- | --- | --- |
| Step | primer | sequence (5’-3’) |
| reverse transcription | oligo-dT adapter primer | (included in a TaKaRa kit) |
| 1^st^ PCR | adapter primer  N3-LC1 | (included in a TaKaRa kit)  GCAGTGTAGAGGAATACGGA |
| 2^nd^ PCR | adapter primer  N3-LC | (included in a TaKaRa kit)  AATACGGAGAGCTGTCAAAA |
| 5’ RACE |  |  |
| Step | primer | sequence (5’-3’) |
| reverse transcription | N3-AN | ACTAAAGAAAGCCCTGTTCTTT |
| 1^st^ PCR | AUAP  N3-LN | (included in a RACE kit)  GAGAGAAATAATGGGAACAC |
| 2^nd^ PCR | AUAP  N3-LN1 | (included in a RACE kit)  GCTAATACCAAGTTTCTGAAACT |
