# Supplementary material for: Sense Transgene-Induced Post-Transcriptional Gene Silencing in Tobacco Compromises the Splicing of Endogenous Counterpart Genes
Source: PLoS One. 2014 Feb 21;9(2):e87869. doi: 10.1371/journal.pone.0087869 (PMC3931610; doi:10.1371/journal.pone.0087869)
Supplement: Table S3 — List of primers used for the data in Figure 2 . (DOC) [file pone.0087869.s007.doc]

| qRT-PCR assay |  |  |
| --- | --- | --- |
| amplified region: e1-e2 (356 bp) | |  |
| step | primer | sequence (5’-3’) |
| reverse transcription | NFAD3-N1 | CATGTCCAAGAACAAAGATTGCC |
| PCR | N3-LC1 | GCAGTGTAGAGGAATACGGA |
|  | NFAD3-N1 | CATGTCCAAGAACAAAGATTGCC |
| amplified region: e8-e9 (494 bp) | |  |
| step | primer | sequence (5’-3’) |
| reverse transcription | N3-AN | ACTAAAGAAAGCCCTGTTCTTT |
| PCR | N3-Ce8 | GAGGAGGACTAACCACAGTT |
|  | N3-AN | ACTAAAGAAAGCCCTGTTCTTT |
| amplified region: e1-e7 (816 bp) | |  |
| step | primer | sequence (5’-3’) |
| reverse transcription | N3-AN | ACTAAAGAAAGCCCTGTTCTTT |
| PCR | N3-LC2 | GTGTAGAGGAATACGGAGAGCTGTC |
|  | N3-Ne6/7 | CCAGCCACATTACAAAAATCAAGT |
| ChiP-qPCR assay |  |  |
| amplified region | primer | sequence (5’-3’) |
| *endo-NtFAD3* 5’region | ChIP-N3-Fw | GAGGAATACGGAGAGCTGTCAAA |
| (332 bp) | ChIP-N3-Rv | CACCATTTTCCCATCTCAGAA |
| *endo-NtFAD3* 3’ region | N3-AN | ACTAAAGAAAGCCCTGTTCTTT |
| (250 bp) | N3-C2 | CCAGTACTTGGCAAGTATTA |
| *NtFAD7* 3’ region | ChIP-N7-Fw | TCAATGCAATATGCGATGGT |
| (223 bp) | ChIP-N7-Rv | ACCCTGAGAGTTGGGGATCA |
| *EF1α* | EF-1α-F | TGAGATGCACCACGAAGCTC |
| (51 bp) | EF-1α-R | CCAACATTGTCACCAGGAAGTG |
|  |  |  |
