# Supplementary material for: Sense Transgene-Induced Post-Transcriptional Gene Silencing in Tobacco Compromises the Splicing of Endogenous Counterpart Genes
Source: PLoS One. 2014 Feb 21;9(2):e87869. doi: 10.1371/journal.pone.0087869 (PMC3931610; doi:10.1371/journal.pone.0087869)
Supplement: Table S4 — List of primers used for the deep sequencing of nuclear RNAs. (DOC) [file pone.0087869.s008.doc]

| Amplified gene | primer | sequence (5’-3’) |
| --- | --- | --- |
| *endo-NtFAD3* | N3-AN | ACTAAAGAAAGCCCTGTTCTTT |
| *NtFAD7* | N7-LN | GTGATTACAAACTTGAATGGCC |
| *EF-1α* | NtEF1-a-Rv1 | GCCTCTTGGGCTCATTAATC |
